# CovNet: A Transfer Learning Framework for Automatic COVID-19 Detection From Crowd-Sourced Cough Sounds

**DOI:** 10.3389/fdgth.2021.799067

**Published:** 2022-01-03

**Authors:** Yi Chang, Xin Jing, Zhao Ren, Björn W. Schuller

**Affiliations:** ^1^Group on Language, Audio, and Music, Imperial College London, London, United Kingdom; ^2^Chair of Embedded Intelligence for Health Care and Wellbeing, University of Augsburg, Augsburg, Germany; ^3^L3S Research Center, Hannover, Germany

**Keywords:** transfer learning, COVID-19, cough, FluSense, COUGHVID

## Abstract

Since the COronaVIrus Disease 2019 (COVID-19) outbreak, developing a digital diagnostic tool to detect COVID-19 from respiratory sounds with computer audition has become an essential topic due to its advantages of being swift, low-cost, and eco-friendly. However, prior studies mainly focused on small-scale COVID-19 datasets. To build a robust model, the large-scale multi-sound FluSense dataset is utilised to help detect COVID-19 from cough sounds in this study. Due to the gap between FluSense and the COVID-19-related datasets consisting of cough only, the transfer learning framework (namely CovNet) is proposed and applied rather than simply augmenting the training data with FluSense. The CovNet contains (i) a parameter transferring strategy and (ii) an embedding incorporation strategy. Specifically, to validate the CovNet's effectiveness, it is used to transfer knowledge from FluSense to COUGHVID, a large-scale cough sound database of COVID-19 negative and COVID-19 positive individuals. The trained model on FluSense and COUGHVID is further applied under the CovNet to another two small-scale cough datasets for COVID-19 detection, the COVID-19 cough sub-challenge (CCS) database in the INTERSPEECH Computational Paralinguistics challengE (ComParE) challenge and the DiCOVA Track-1 database. By training four simple convolutional neural networks (CNNs) in the transfer learning framework, our approach achieves an absolute improvement of 3.57% over the baseline of DiCOVA Track-1 validation of the area under the receiver operating characteristic curve (ROC AUC) and an absolute improvement of 1.73% over the baseline of ComParE CCS test unweighted average recall (UAR).

## 1. Introduction

Since the year 2019, the coronavirus disease 2019 (COVID-19) caused by severe acute respiratory syndrome coronavirus 2 (SARS-CoV-2) has become a global pandemic^1^. As of August 2021, there have been more than 202, 000, 000 confirmed cases of COVID-19 worldwide, including more than 4, 000, 000 deaths, reported by the World Health Organization (WHO)^2^. The daily ^1^ ^2^ increasing COVID-19 cases and deaths have resulted in global lockdown, quarantine, and many restrictions (1). Along with the above measures, a set of following problems have appeared, including the economic downturn (2) and mental health problems (e.g., depression and stress) (1).

Swift and accurate diagnosis of COVID-19 is essential to give patients appropriate treatments and effectively control its transmission (3). The reverse transcription PCR (RT-PCR) from oral-nasopharyngeal swabs identifies viral RNA and is a commonly used instrument for the diagnosis of COVID-19. Nevertheless, high false negative rate and stability issues have been reported (4). In contrast to RT-PCR, chest CT was proven to have high sensitivity and be expedited for diagnosing COVID-19(4). Serological instruments are utilised to diagnose/confirm late COVID-19 cases by measuring antibody responses to the corresponding infection (5). Compared to the above laboratory instruments, which require professionals and special medical equipment, rapid antigen and molecular tests using nasopharyngeal swabs are commercially available due to their swift and simple test procedures, reduced mortality of COVID-19 patients, internal hospital costs, and in-hospital transmission (6). However, rapid tests are still hard-to-follow for non-specialists and are not environment-friendly.

Artificial intelligence has been widely applied to respiratory sounds in the healthcare area (7–9). In a study by (8), a multilayer perceptron based classifier was developed on features extracted from respiratory sounds to screen lung health. Random forests are applied on the filter bank energy-based features to pre-screen the lung health abnormalities (9). COVID-19 patients were reported to have seven common symptoms, including fever, cough, sore throat, headache, myalgia, nausea/vomiting, and diarrhea (10). Among these symptoms, the first two symptoms of COVID-19 are fever and cough (10). As a fast and non-invasive way to detect potential infections in public areas, body temperature measurement has been commonly employed (11). Traditional body temperature measurement with a thermometer usually requires relatively close contact with potential COVID-19 positive individuals (12). Although infrared (IR) thermal cameras provide a non-contact way for mass fever detection, they may not be valid because of the absence of calibration, non-homogeneous devices/protocols, and poor correlation between skin temperature and core body temperature (11). The reading of IR thermal cameras could also be affected by the environmental temperature (11). On the other hand, cough, as a common symptom in many respiratory diseases, is a worthwhile consideration when diagnosing a disease (13). Cough sounds have been used to diagnose asthma, bronchitis, pertussis, pneumonia, etc. (13). Recent studies have also investigated the feasibility of detecting COVID-19 infections from cough sounds. For instance, cough sounds were shown to contain latent features distinguishable between COVID-19 positive individuals and COVID-19 negative individuals (i.e., normal, bronchitis, and pertussis) (14). In Brown et al.'s study (15), cough sounds from COVID-19 positive individuals were reported to have a longer duration, more onsets, higher periods, lower RMS, and MFCC features with fewer outliers. Due to the development of the internet-of-things (IoT), the algorithms for detecting potential COVID-19 positive individuals from cough sounds can be integrated into mobile phones, wearable devices, and robots. Such a rapid, easy-to-use, and environment-friendly instrument will be helpful for real-time and remote pre-screening of COVID-19 infections, thereby supplementing clinical diagnosis and reducing the medical burden.

Since the outbreak of COVID-19, several studies have collected cough samples from COVID-19 positive patients (and COVID-19 negative individuals) to detect COVID-19 infections. Coswara (16) is a crowd-sourced database consisting of various kinds of sounds, including breathing (shallow and deep), coughing (shallow and deep), sustained vowel phonation (/ey/ as in made, /i/ as in beet, /u:/ as in cool), and number counting from one to twenty (normal and fast-paced). Another crowd-sourced database, COUGHVID with cough sounds only (17), was collected via a web interface. To date, the latest version of COUGHVID is publically released with 27, 550 cough recordings.^3^ The crowd-sourced University of Cambridge COVID database was reported to have more than 400 cough and breathing recordings (15). The Virufy datasets consist of a Latin American crowd-sourced dataset (31 individuals) and two South Asian clinical datasets (362 and 63 individuals, respectively). Due to the difficulty of collecting cough sounds of confirmed COVID-19 patients and multi-sound (non-cough)/noise in crowd-sourced datasets, most of the above databases are small-scale, leading to a challenge for training robust machine learning models.

**Figure 1:**
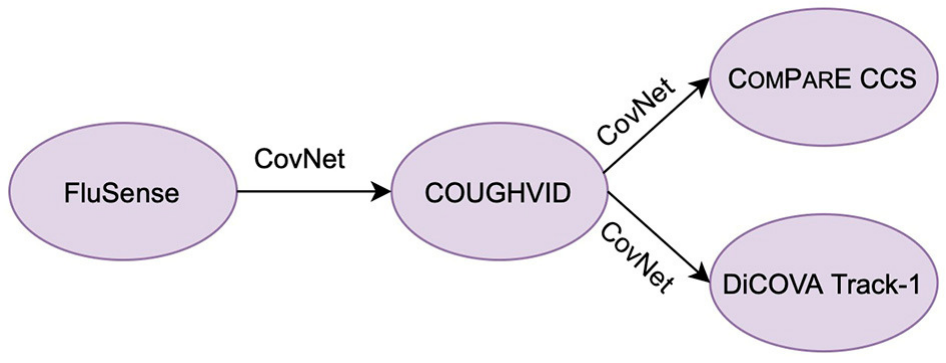
The workflow of this study. CovNet is the proposed transfer learning framework, which includes transferring parameters and incorporating embeddings. CovNet is first applied on the Flusense as the source data, COUGHVID as the target data. Afterwards, to further validate the effectiveness of CovNet, the CovNet based pre-trained COUGHVID models are applied on two smaller Computational Paralinguistics challengE (ComParE) 2021 COVID-19 cough sub-challenge (CCS) dataset and DiCOVA 2021 Track-1 dataset.

With this in mind, we propose a hybrid transfer learning framework for robust COVID-19 detection, where several convolutional neural networks (CNNs) are trained on large-scale databases and fine-tuned on several small-scale cough sound databases for verification. Note that the focus of this paper is not to outperform the state-of-the-art neural networks models for COVID-19 detection from cough sounds; rather, the aim of this study is to provide a framework for mitigating the effect of noise or irrelevant sounds in the crowd-sourcing datasets applied to COVID-19 by training robust CNN models with the transferred knowledge from Flusense and/or COUGHVID. The workflow of this study is indicated in Figure 1. The code of this paper is publicly available on GitHub^4^.

- The FluSense database (18) was collected in a platform to track influenza-related indicators, such as cough, sneeze, sniffle, and speech. Since it contains various types of sounds existing in crowd-sourced cough datasets, the FluSense dataset is applied in this study.
- Due to the gap in sound type between FluSense and databases with cough sounds only, the COUGHVID database is considered as the target data when CNNs are trained on FluSense as the source data. The trained models on COUGHVID are further adapted to the other two smaller test databases, i.e., Computational Paralinguistics challengE (ComParE) 2021 COVID-19 cough sub-challenge (CCS) (19) and DiCOVA 2021 Track-1 (20).
- We propose two transfer learning pipelines, i.e., transferring parameters from the source database to the target database for fine-tuning models and incorporating embeddings for expanding models' capability of extracting useful features.

In the following sections, the transfer learning framework is first introduced in section 2, followed by the architecture of the models for COVID-19 detection in section 3. Next, the experimental details are described, and the results are presented and discussed in section 4. Finally, our study is summarised, and the outlook is given in section 5.

## 2. Transfer Learning Frameworks

Transfer learning aims at applying the knowledge learnt from source data to different but related target data and achieving better performance in a cost-effective way (21–23). The source data and target data should be similar, otherwise negative transfer may happen (22, 24). Transfer learning has been successfully applied to COVID-19 detection based on acoustic data (14, 15). In Imran et al.'s study (14), the knowledge was transferred from the cough detection model to the COVID-19 diagnosis model. Brown et al. (15) discovered that VGGish pre-trained on a large-scale YouTube dataset was utilised to extract audio features from raw audio samples for COVID-19 diagnosis.

**Figure 2:**
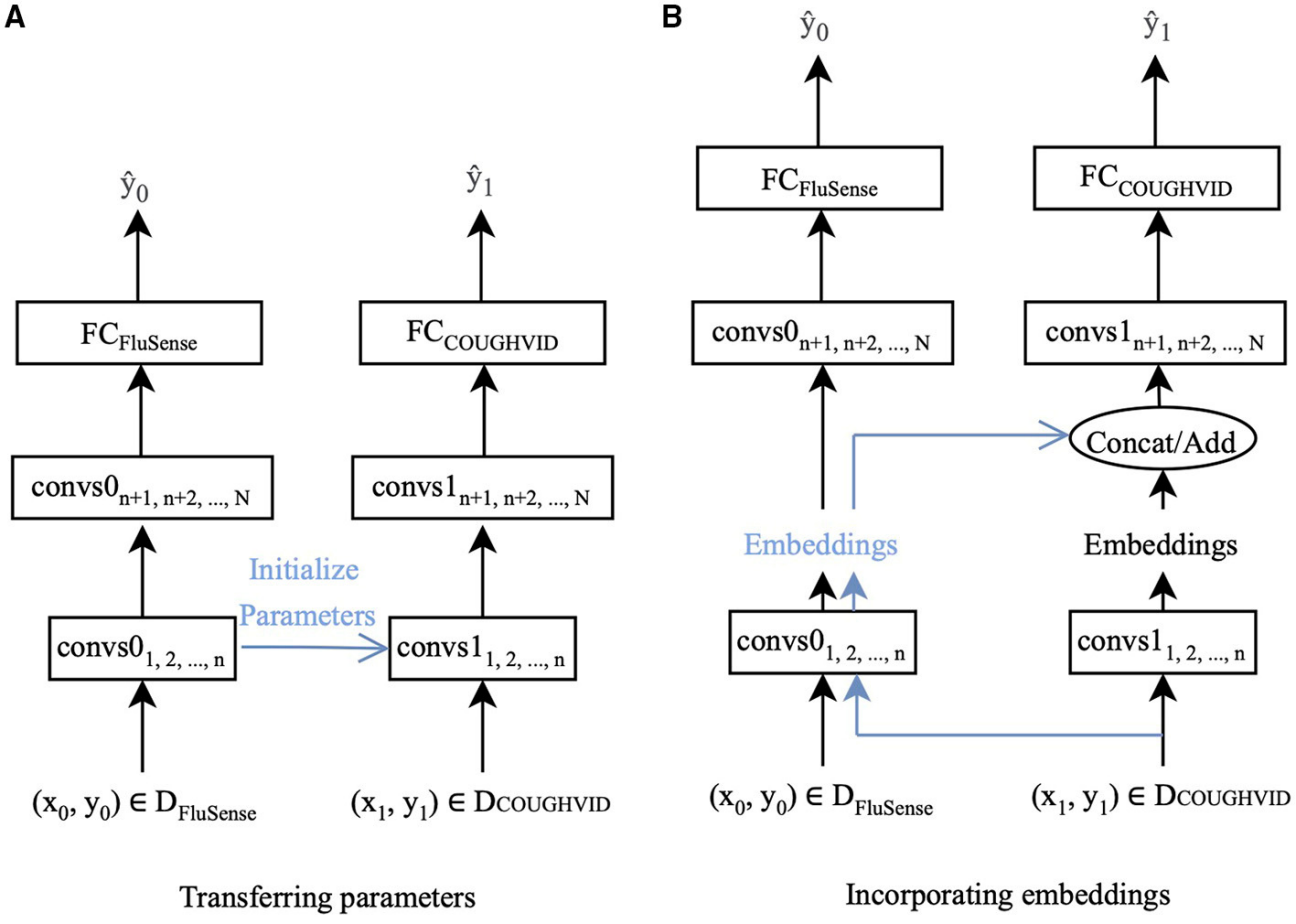
The proposed transfer learning framework :CovNet. **(A)** Parameters of the first *n* convolutional layers/blocks (convs1) of the current COUGHVID model are frozen and initialised by the corresponding first *n* convolutional layers/blocks (convs0) of the pre-trained FluSense model. **(B)** Embeddings are extracted after the *n*-th convs0 of the pre-trained FluSense model. The extracted embeddings are concatenated or added to the current embeddings generated after the *n*-th convs1 of the COUGHVID model.

In this study, two ways of transfer learning are applied. One is to fine-tune the parameters of the networks with the target data. The other is extracting the embeddings from the pre-trained network and applying the embeddings when training the new network for the target dataset. Since the crowd-sourced cough recordings usually contain non-cough audio signals other than cough sounds, such as speech and breathing, the FluSense dataset and the COUGHVID dataset contain similar sound types. Therefore, the knowledge learnt from FluSense data can be employed to improve the performance of models trained on the COUGHDVID dataset. In Figure 2, D_*FluSense*_ is the FluSense dataset, and D_*COUGHVID*_ means the COUGHVID dataset; convs0 and convs1 represent the convolutional layers/blocks in the neural networks on the FluSense dataset and the COUGHVID dataset, respectively; FC_*FluSense*_ and FC_*COUGHVID*_ denotes the fully-connected (FC) layer of corresponding models. When separating the left part with the right part in Figures 2A,B, with the training data (*x*_0_, *y*_0_) and (*x*_1_, *y*_1_), we separately train the CNNs on the FluSense and COUGHVID datasets to produce the predicted values ${\hat{\text{y}}}_{0}$ and ${\hat{\text{y}}}_{1}$, respectively.

With the parameters and embeddings from the pre-trained FluSense models, as highlighted in blue in Figure 2, the COUGHVID models are given the potential to discriminate between the various audio signals, which further helps its COVID-19 detection from crowd-sourced cough signals. Notably, the predicted value ${\hat{\text{y}}}_{1}$ is the final output of the proposed transfer learning framework.

To further investigate the generalisation ability of CovNet, we apply it to some other small-scale crowd-sourced datasets for COVID-19 detection. In the following, we introduce the two transfer learning methods in greater detail.

### 2.1. Transferring Parameters

Fine-tuning pre-trained models is an effective transfer learning method by sharing some parameters across tasks (21, 22). In the computer vision area, parameters of pre-trained models on ImageNet (25) are often applied for transfer learning on a wide range of image-related tasks (26–29). Similarly, parameters of pre-trained models on the Audio Set are transferred to many audio-related tasks (30–32). Parameters of pre-trained CNN models on the Audio Set are transferred to the adapting networks for acoustic event recognition (30, 31). Several pre-trained audio neural networks trained on the Audio Set dataset were proposed for other audio pattern recognition tasks (32).

In this study, as indicated in Figure 2A, the parameters of the first *n* convolutional layers/blocks, convs1_1, 2,..., *n*_, of models trained on the COUGHVID dataset, are initialised by the corresponding layers/blocks convs0_1, 2,..., *n*_ of models pre-trained on FluSense dataset. The parameters of convs1_1, 2,..., *n*_ are frozen and not trained, and only the remaining randomly initialised parameters of convs1_*n*+1, *n*+2,..., *N*_ and FC_*COUGHVID*_ are updated during the training procedure.

### 2.2. Incorporating Embeddings

The embeddings generated by the convolutional layers carry either low-level edge information or high-level discrimination-related features (22, 23). Moreover, the performance of embeddings appears to be highly scalable with the amount of training data (33). In this study, the pre-trained FluSense models produce embeddings representing high-level or low-level characteristics of various audio types, which can be applied as an additional input to help develop the target model.

Specifically, we feed the crowd-sourced cough recordings from the COUGHVID into the pre-trained Flusnese model and extract the embeddings after certain convolutional layers/blocks. Figure 2B exhibits this strategy. Data-point (*x*_1_, *y*_1_) enters the pre-trained FluSense model, and the output embeddings of the *n*-th convolutional layer/block convs0_*n*_ are extracted to be concatenated (on the channel dimension) or added with the embeddings generated by the corresponding convs1_*n*_. The concatenated or added embeddings enter the next convolutional layer/block convs1_*n*+1_ for the task of COVID-19 detection.

## 3. Automatic COVID-19 Detection

Convolutional neural networks have been successfully applied in image-related areas, such as image classification (34–37). When processing audio signals, CNNs have demonstrated their capabilities in extracting effective representations from the log Mel spectrograms (38, 39). In this study, we choose four typical CNN models: base CNN (34), VGG (40), residual network (ResNet) (41), and MobileNet (42). We focus on the proposed transfer learning framework, CovNet, instead of competing with the state-of-the-art models on COVID-19 detection. Therefore, in order to highlight the effectiveness of CovNet, we construct four simple CNN models (i.e., CNN-4, VGG-7, ResNet-6, and MobileNet-6), each of which only has three convolutional layers/blocks. A detailed description of each model is given and analysed in the following subsections.

The log Mel spectrograms are calculated by Mel filter banks and logarithmic operation worked on the spectrograms, which are produced by the Short-Time Fourier Transforms (STFTs) on the original waveforms. In this section, to better evaluate the effectiveness of the proposed transfer learning framework and compare the performance differences among different CNN architectures, four CNNs are employed to deal with the extracted log Mel spectrograms: CNN-4, VGG-7, ResNet-6, and MobileNet-6. Log Mel spectrograms (*T*,*F*) are extracted from the audio signals as the input to the CNNs, where *T* represents the sequence length, and *F* denotes the log Mel frequency. Before entering the final FC layer, the matrix has the dimension (*C*_*N*_, *N*), where *C*_*N*_ is the output channel number of the last convolutional layer, and *N* is the class number. Specifically, for the FluSense database, *N* is set to be 9; for the other datasets used in this study, *N* equals 2. For comparison convenience, we regard the convolutional layers and blocks equally when ordering them in a specific model. In this notation, ResNet-6 and MobileNet-6 have “block2” following the first convolutional layer.

### 3.1. CNN-4

**Figure 3:**
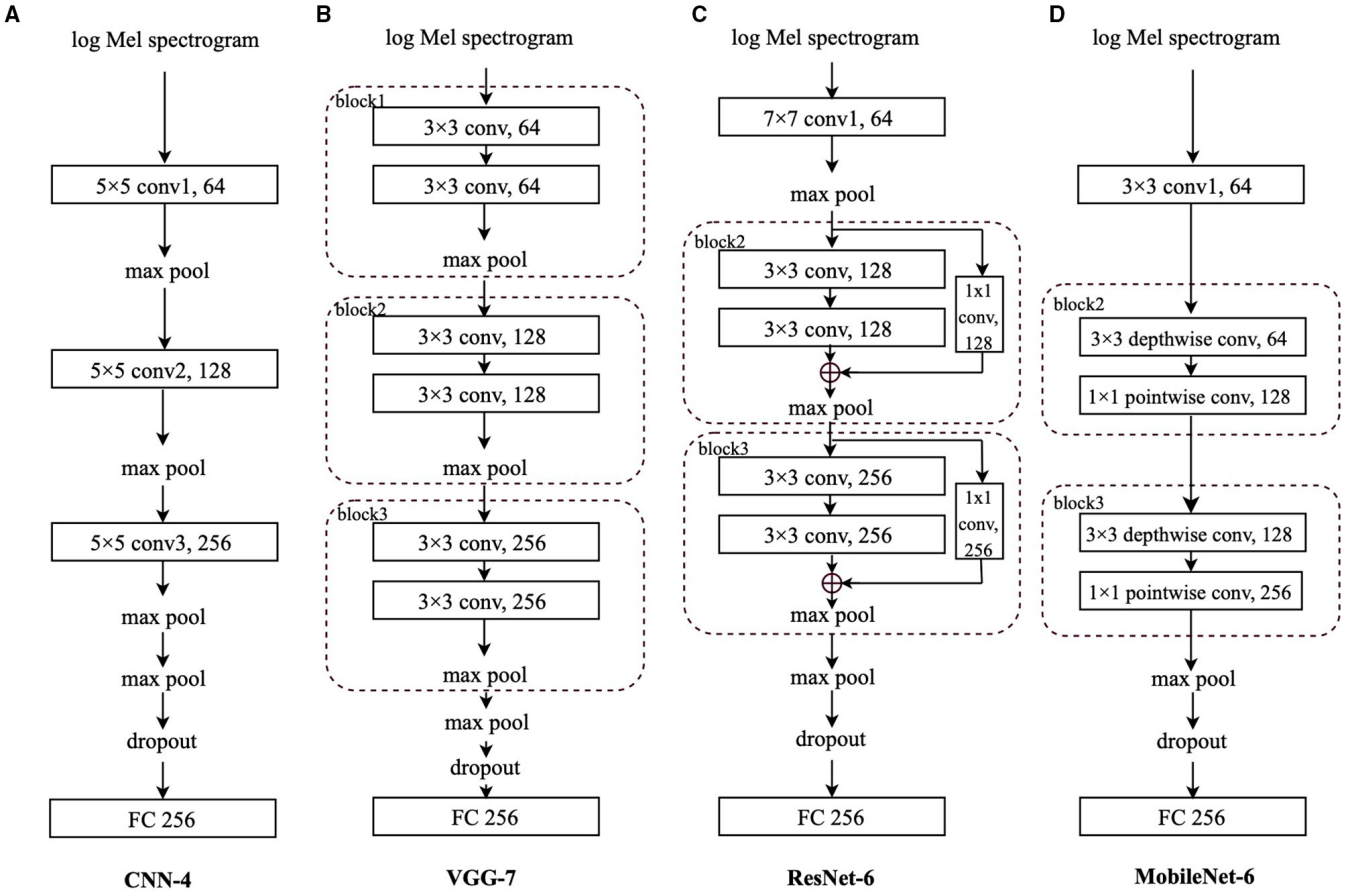
Models' architecture: **(A)** Convolutional neural network-4 (CNN-4), **(B)** VGG-7, **(C)** residual network-6 (ResNet-6), **(D)** MobileNet-6. “conv” stands for the convolutional layer, and “block” indicates the convolutional block. The number before the “conv” is the kernel size; the number after the “conv” is the output channel number. The number after “FC” is the input neurons' size.

As shown Figure 3A, we propose a simple 4-layer CNN, CNN-4, constructed by three 5 × 5 convolutional layers. To speed up and stabilise the training procedure, each convolutional layer is followed by batch normalisation (43) and the Rectified Linear Unit (ReLU) activation function (44). Afterwards, we apply max pooling for downsampling. The first three local max pooling operations are conducted over a 2 × 2 kernel, and the last max pooling is a global one to summarise the features along the dimension of the sequence length and frequency. Before the final FC layer for the final predicted result, a dropout (45) layer is utilised to address the overfitting issue.

### 3.2. VGG-7

Very deep CNN, known as VGG, were originally designed with up to 19 weight layers and achieved great performance on the large-scale image classification task (40, 46). VGG or VGG-like architectures were applied to extract audio features from respiratory sound data for COVID-19 detection and obtained good performances (15, 47).

As indicated in Figure 3B, we adapt the VGG (40) with 7 layers, VGG-7, which is composed of three convolutional blocks and a final FC layer. Although the VGG-7 is simple, different from its original “deep” design, it is still worthwhile to include it for fair comparison with other CNNs in this study. Each block contains two 3 × 3 convolutional layers, each of which is followed by batch normalisation (43) and the ReLU function (44) to stabilise and speed up the training process. Afterwards, a local max pooling layer with a kernel size of 2 × 2 is applied. Following the three blocks, there is also a global max pooling layer working on the sequence length and log Mel frequency dimensions. Before the FC layer, a dropout (45) layer is applied.

### 3.3. ResNet-6

The Deep ResNet is proposed to address the degradation problem existing in training deeper networks (41) by incorporating shortcut connections between convolutional layers. In Hershey et al.'s (48) study,ResNet has outperformed other CNNs for audio classification on the Audio Set (49). A ResNet based model is constructed for COVID-19 detection from breath and cough audio signals (50).

In this study, we mainly adopt the above mentioned shortcut connections to construct a 6-layer ResNet, ResNet-6. In Figure 3C, after the first convolutional layer with a kernel size of 7 × 7 followed by batch normalisation (43) and the ReLU function (44), we apply two convolutional blocks, each of which contains the “shortcut connections” to add the identity mapping with the outputs of two stacked 3 × 3 convolutional layers.

Inside “block2” and “block3,” after the first 3 × 3 convolutional layer, the batch normalisation (43) and ReLU function (44) are applied, whereas only the batch normalisation is utilised after the second 3 × 3 convolutional layer. For the channel number consistency, the identity is processed by a 1 × 1 convolutional layer followed by batch normalisation (43); after the addition of the identity and the output of two stacked convolutional layers, we apply the ReLU function (44). The max pooling after the 7 × 7 convolutional layer is a local one with a kernel size of 3 × 3 and the max pooling layers in “block2” and “block3” are also local with a kernel size of 2 × 2; similarly, the last max pooling is a global one, followed by a dropout (45) layer and the FC layer.

### 3.4. MobileNet-6

Based on depthwise separable convolutions, light-weight MobileNets have been widely applied in mobile and embedded image related applications (42, 51). MobileNets are cost-effective and are explored herein for potential solutions embedded in mobile devices for COVID-19 detection.

We adapt the MobileNet with 6 layers only. As shown in Figure 3D, after the first 3 × 3 convolutional layer followed by batch normalisation (43) and the ReLU function (44), each of “block2” and “block3” contains a 3 × 3 depthwise convolutional layer and a 1 × 1 pointwise convolutional layer, respectively. Similarly, batch normalisation (43) and ReLU function (44) are applied after each convolutional layer. Similar to the original MobileNet architecture, we only set one global max pooling layer before the dropout (45) layer and the final FC layer.

## 4. Experimental Results

With the aforementioned transfer learning framework, the experiments will be presented in this section, including the databases, experimental setup, results, and discussions.

### 4.1. Databases

To verify the proposed transfer learning framework in this study, the following four datasets are employed.

#### 4.1.1. FluSense

The FluSense (18) project applied a part of the original Audio Set dataset (49), which includes weakly labelled 10-s audio clips from YouTube. After the re-annotation by two human raters for more precise labels in the FluSense (18) project, there are a total of 45, 550 seconds samples in Audio Set that are considered in this study, and they are labelled with the classes of *breathe, burp, cough, gasp, hiccup, other, silence, sneeze, sniffle, snore, speech, throat-clearing, vomit*, and *wheeze*. To mitigate the effect of data imbalance on the classification performance, those classes with a number of samples less than 100 are not considered in our experiments. Therefore, the audio samples labelled with the following nine classes are employed: *breathe, cough, gasp, other, silence, sneeze, sniffle, speech*, and *throat-clearing*. For all audio recordings in the above nine classes, we first re-sampled them into 16 kHz. Second, as the audio samples have various time lengths, we split the original samples with a length of greater than or equal to 0.5 s into one or more 1 s segment(s). In particular, for audio samples with a length between 0.5 and 1 s, the audio repeats itself until a full 1 s segment is reached. For those samples with a length greater than 1 s, after a certain number of 1 s segments are split, the remaining signals repeat themselves until a full segment is reached if the remaining one has a length of greater than or equal to 0.5 s; otherwise, the remaining signals are simply abandoned. Furthermore, we split the segments into train/val subsets with a ratio of 0.8/0.2 in a stratified manner. The data distribution of FluSense before and after the pre-processing is shown in Table 1.

**Table 1:** Data distribution of the FluSense data.

	**Original**	**Pre-Processing**		
	**#**	**Train**	**Val**	**∑**
Breathe	167	238	58	297
Cough	2,486	6,148	1,537	7,685
Gasp	337	315	79	394
Other	3,863	15,059	3,765	18,824
Silence	832	1,116	279	1,395
Sneeze	611	540	135	675
Sniffle	589	604	151	755
Speech	2,615	16,614	4,154	20,768
Throat clearing	102	118	29	147
∑	11,602	40,752	10,188	50,940

*The “original” column indicates the number of audio samples; whereas the “pre-processing” columns show the number of segments with unified length of 1 s*.

#### 4.1.2. COUGHVID

The on-going crowd-sourced COUGHVID dataset (17) is collected via a web interface^5^. All participants voluntarily record and upload their cough sounds lasting for up to 10 s. In the meantime, the COVID-19 status of each cough sample is self-reported by each participant: *healthy, symptomatic without COVID-19 diagnosis*, and *COVID-19*. The information of each participant is optionally self-reported, including the geographic location (latitude, longitude), age, gender, and whether she/he has other pre-existing respiratory conditions, and muscle pain/fever symptoms. As there might be some low-quality audio samples (e.g., noise, speech, etc.), the data collectors trained an extreme gradient boosting (XBG) classifier on 215 audio samples (121 cough and 94 non-cough) to predict the probability of a recording containing cough sounds. For all audio recordings, the sampling frequency is 48 kHz.

In this study, only the classes of *healthy* (i.e., COVID-19 negative) and *COVID-19* (i.e., COVID-19 positive) are considered, as the audio samples with symptomatic status were not explicitly reported by the participants as to whether they were diagnosed with COVID-19 or not. Furthermore, only audio samples with cough sound probabilities greater than 0.9 are included to ensure each audio sample contains cough sounds. Finally, 7, 774 audio samples (COVID-19 negative: 7, 075, COVID-19 positive: 699) are selected for our experiments. Similarly, we split the selected samples into train/test subsets with a ratio of 0.8/0.2, respectively in a stratified manner. Table 2 shows the data distribution of COUGHVID.

**Table 2:** Data distribution of the COUGHVID data.

**#**	**Train**	**Test**	**∑**
Negative	5,660	1,415	7,075
Positive	559	140	699
∑	6,219	1,555	7,774

#### 4.1.3. ComParE 2021 CCS

In the INTERPSEECH 2021 ComParE (19), the CCS provides a dataset from the crowd-sourced Cambridge COVID-19 Sound database (15). The participants are asked to provide one to three forced coughs in each recording via one of the following multiple platforms: A web interface, an Android app, and an iOS app.^6^ The CCS dataset consists of 929 cough recordings (1.63 h) from 397 participants. The data distribution of CCS is shown in Table 3. All recordings from the CCS dataset were resampled and converted into 16 kHz. The official training, validation, and test sets in the ComParE challenge are used in this study.

**Table 3:** Data distribution of the Computational Paralinguistics challengE (ComParE) COVID-19 cough sub-challenge (CCS) data.

**#**	**Train**	**Val**	**Test**	**∑**
Negative	215	183	169	567
Positive	71	48	39	158
∑	286	231	208	725

#### 4.1.4. DiCOVA 2021 Track-1

The Track-1 of the DiCOVA challenge 2021 (20) provides cough recordings from 1, 040 participants (COVID-19 negative: 965, COVID-19 positive 75). In the challenge, the dataset was split into five train-validation folds. Each training set consists of 822 cough samples (COVID-19 negative: 772, COVID-19 positive: 50), and each validation set contains 218 cough samples (COVID-19 negative: 193, COVID-19 positive: 25). The additional test set is not used in this study, as it is blind. All cough recordings are sampled at 44.1 kHz. The data distribution of DiCOVA 2021 Track-1 is indicated in Table 4.

**Table 4:** DiCOVA Track-1 data distribution of each fold of cross-validation.

**#**	**Train**	**Val**	**∑**
Negative	772	193	965
Positive	50	25	75
∑	822	218	1040

### 4.2. Experimental Setup

For faster progress (38), all audio files in the four datasets are re-sampled into 16 kHz. The log Mel spectrograms are extracted with a sliding window size of 512, an overlap of 256 units, and 64 Mel bins.

As for the evaluation metrics, we mainly use unweighted average recall (UAR), since it is more adequate for evaluating the classification performance on imbalanced datasets than accuracy,—the weighted average recall (52, 53). Apart from the UAR, we also calculate the area under the receiver operating characteristic curve (ROC AUC) score.

The proposed CNNs consist of three convolutional layers/blocks. The number of output channels for the three convolutional layers/blocks is 64, 128, and 256, respectively. During the training procedure of the neural networks, the cross-entropy loss is utilised as the loss function. To overcome the class imbalance issue, we re-scale the weight parameter for each class in the loss function. Since this study focuses on the transfer learning framework, we do not further mitigate the class imbalance issue through down-/up-sampling.

For single learning (i.e., training from scratch) on the FluSense and the COUGHVID datasets, the optimiser is set to “Adam” with an initial learning rate of 0.001, which is scheduled to be reduced by a factor of 0.4 when there is less than 0.01 improvement of the UAR after every 4 of 30 epochs in total. When transferring parameters, we set the initial learning rate as 0.0001; for incorporating embeddings, the initial learning rate is set to be 0.001.

When applying the strategy of transferring parameters introduced in section 2.1 to training the COUGHVID model, we experiment with only setting the following layer(s) trainable: the FC layer, the convolutional layer/block (conv/block) 3 & FC layer, conv/block 2−3 & FC layer, and conv/block 1−3 & FC layer, respectively. The remaining layer(s)/block(s) are initialised based on the pre-trained FluSense models' corresponding parameters and are frozen during the whole training procedure. As for the incorporating embeddings strategy described in section 2.2, we investigate the concatenation and addition of two embeddings generated from the conv/block 3, conv/block 2, and conv/block 1, respectively. One embedding is from the pre-trained FluSense model, and the other one is the COUGHVID model trained from scratch.

To further validate the effectiveness of the CovNet, we apply the pre-trained COUGHVID models on the ComParE CCS dataset and the DiCOVA Track-1 dataset. Specifically, we train the four CNNs introduced in section 3 from scratch. Afterwards, we choose up to two COUGHVID models with the best performance (best AUC or best UAR) as the pre-trained models. With the chosen pre-trained COUGHVID models and their strategies (layer(s)/block(s) number and transfer learning strategies), we transfer the parameters or embeddings of the above chosen COUGHVID models to the current train-from-scratch models on the ComParE and DiCOVA datasets during the training. Finally, we choose the best results to compete with official baselines: the average validation AUC 68.81% (20) for the DiCOVA Track-1 dataset, and test UAR without fusion 64.7% (19) for ComParE CCS. Similarly, when training models from scratch or applying the incorporating embeddings method, we set the initial learning rate as 0.001, whereas if the transferring parameters are utilised, the initial learning rate is set as 0.0001.

### 4.3. Results

In Table 5, we focus on performance differences on the COUGHVID test dataset between single learning (training from scratch) models and the models produced by the proposed transfer learning strategies in section 2. For convenience, the best test AUC and test UAR of every model under three transfer learning strategies are shown in bold face. We can see that there are some improvements in test AUC/UAR, especially for the VGG-7 and MobileNet-6. In the following analysis, we compare the absolute difference between performances. On the COUGHVID test dataset, with the transfer learning, the VGG-7 obtains an improvement of 2.62% AUC (*p* < 0.1 in a one-tailed *z*-test) and an improvement of 3.75% UAR (*p* < 0.05 in a one-tailed *z*-test); the MobileNet-6 achieves 3.77% improvement in AUC (*p* < 0.05 in a one-tailed *z*-test) and 4.88% improvement in UAR (*p* < 0.005 in a one-tailed *z*-test). Moreover, for all constructed CNN models, only setting the FC layer trainable and freezing other layers with parameters transferred from pre-trained FluSense models achieves almost the lowest AUC/UAR among all transfer learning settings.

**Table 5:** Models' performances [AUC/UAR %] on FluSense and COUGHVID test datasets.

		**Layers**	**CNN-4**	**ResNet-6**	**VGG-7**	**MobileNet-6**
Single Learning	FluSense	—	93.55/65.27	93.91/64.76	93.23/63.86	91.26/58.24
	COUGHVID	—	66.14/59.43	68.86/60.43	65.15/56.42	64.17/54.83
Transfer Learning	Parameters	FC	58.59/53.68	61.35/57.50	54.68/54.14	56.91/53.93
		conv/block 3 & FC	68.04/57.04	67.01/57.97	64.97/57.15	67.88/**59.71**
		conv/block 2-3 & FC	69.05/**60.98**	**67.89**/**59.25**	64.92/**59.79**	**67.94**/58.93
		conv/block 1-3 & FC	**69.43**/55.54	66.23/56.31	**67.31**/56.17	65.21/55.64
	Embeddings Cat	conv/block 3	**67.73**/**60.65**	**67.21**/59.45	**65.85**/**58.27**	64.32/**56.46**
		conv/block 2	67.30/57.81	66.17/55.59	65.58/52.30	**67.36**/52.31
		conv/block 1	65.15/59.30	65.35/**59.77**	58.67/51.92	66.37/53.77
	Embeddings Add	conv/block 3	**66.76**/**59.30**	64.27/**58.88**	66.08/**60.17**	**65.94**/**58.24**
		conv/block 2	66.39/58.82	64.55/57.27	**67.77**/58.55	64.37/57.19
		conv/block 1	65.91/57.17	**64.63**/58.21	63.85/58.97	64.17/56.60

*Single learning indicates training from scratch and transfer learning includes “Parameters” (transferring parameters), “Embeddings Cat,” and “Embeddings Add” (incorporating emebdddings). The Models' performances with transfer learning are based on the COUGHVID dataset. For “Parameters,” the “Layers” column indicates the layers that are randomly initialised and trainable during the training procedure, and the remaining layers are frozen and initialised by the pre-trained FluSense models; for “Embeddings Cat,” “Embeddings Add,” and “Layers,” the column lists the convolutional layer/block (conv/block), after which embeddings incorporation happens. For convenience, the best test AUC and test UAR of every model under three transfer learning strategies are shown in bold face*.

For the transferring parameters strategy, we can see that most best test AUC/UAR cases are obtained by only setting the convolutional layer/block (conv/block) 2−3 & FC layer trainable or the conv/block 1−3 & FC layer trainable. With the embeddings cat method, models' performances are mostly better than single learning models' and the most best results are achieved by concatenating the embeddings output by the conv/block 3. With the embeddings addition method, models also mostly outperform the single learning ones, and similarly, most best results are obtained by adding embeddings after the conv/block 3.

In Table 6, first, we can see that with the proposed transfer learning strategies on the pre-trained COUGHVID models generated by the CovNet, most of the models' performances improve a lot compared with the single learning models' performance. Specifically, transferring parameters improves the test UAR on ComParE by 9.05% for the VGG-7 (*p* < 0.05 in a one-tailed *z*-test); the transferring parameters improves the validation AUC on DiCOVA by 1.12, 3.86, and 5.22 % for the CNN-4, ResNet-6, and VGG-7, respectively (in a one-tailed *z*-test, not significant, *p* < 0.05, and *p* < 0.005, respectively). The incorporating embeddings improves the test UAR on ComParE data by 1.47, and 1.11% for the CNN-4, and VGG-7, respectively; the incorporating embeddings improves the validation AUC of DiCOVA by 3.62%, 8.85, 7.46, and 2.20% for the CNN-4, ResNet-6, VGG-7, and MobileNet-6, respectively (in a one-tailed *z*-test, *p* < 0.05, *p* < 0.001, *p* < 0.001 and not significant, respectively).

**Table 6:** Models' performances [ %], validation AUC on the DiCOVA Track-1 dataset, and test UAR on the ComParE dataset, with single learning (train from scratch), and the proposed transfer learning strategies.

		**Dataset**	**Baseline**	**CNN-4**	**ResNet-6**	**VGG-7**	**MobileNet-6**
Single Learning	–	ComParE	64.70	63.35	61.78	57.38	63.80
		DiCOVA	68.81	68.76	62.53	64.88	64.27
Transfer Learning	Parameters	ComParE	–	61.24	60.01	**66.43**	57.22
		DiCOVA	–	**69.88**	66.39	**70.10**	63.29
	Embeddings	ComParE	–	**64.82**	60.67	58.49	63.37
		DiCOVA	–	**72.38**	**71.38**	**72.34**	66.47

*Pre-trained COUGHVID models and their corresponding transfer learning settings are chosen based on the best performance in Table 5. “Embeddings” here include addition/concatenation. The numbers in bold are higher than the baseline*.

**Figure 4:**
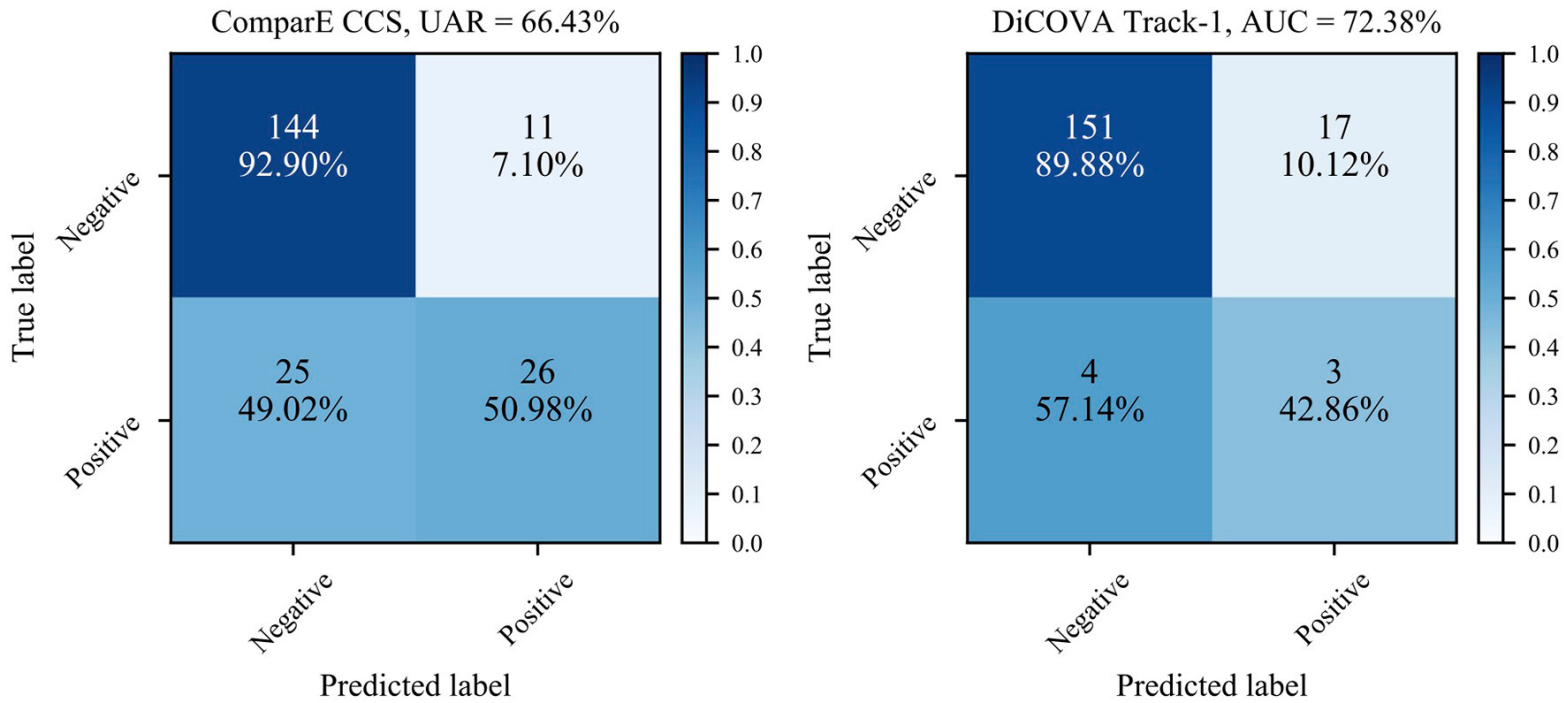
Confusion matrices for the best performance on the ComParE CCS test set and the DiCOVA validation set. For the DiCOVA dataset, since its test dataset is not accessible, the numbers are averaged over the five cross-validation folds.

Second, as the numbers in bold indicate better performance than the baseline, we can see that most models learnt through the transfer learning framework outperform the official baselines, even though the models here are quite simple. Notably, the best test UAR 66.43% on ComParE CCS data is achieved by the VGG-7 with transferring parameters, which is 1.73% above the official baseline; the CNN-4 with incorporating embeddings the achieves the best validation AUC 72.38% on the DiCOVA Track-1, which is 3.57% higher than the baseline (*p* < 0.05 in a one-tailed *z*-test). Figure 4 displays the confusion matrices for above-mentioned best UAR on the ComParE CCS dataset and best validation AUC on the DiCOVA Track-1 dataset. We can see that the models recognise negative samples very well, but the positive ones are frequently confused with the negative ones.

### 4.4. Discussion

In Table 5, if comparing the performance of single learning CNNs and transfer learning CNNs, we find that there is no improvement or even slightly worse performance of transfer learning methods on the ResNet-6 model. ResNet gains accuracy from increased neural network depth (41), which may explain the performance of the simple ResNet-6 in this study. Apart from fine-tuning the parameters of FC layers only, almost all other CNN models obtain better performance after the transfer learning, proving the usefulness of the knowledge transferred from the FluSense dataset for recognising COVID-19 on the COUGHVID dataset. Setting FC layers trainable only limits the generalisation of the pre-trained FluSense models.

For fine-tuning parameters of different layers, fine-tuning the weights of the convolutional layers/blocks 2−3 & FC layer obtains better performance. Since the target dataset COUGHVID is not large-scale enough compared with the FluSense one, fine-tuning the entire network (convolutional layers/blocks 1−3 & FC layer) might encounter an overfitting issue (23). Specifically, earlier layers/blocks generate low-level, generic features, which do not change significantly during the training procedure (23). Conversely, the convolutional layer/block 3 herein generates more high-level, domain-dependent representations. As for the embeddings incorporation, concatenation and addition of the embeddings achieve similar results, which indicates that both operations equally transfer the knowledge learnt from the FluSense dataset. Furthermore, we find that incorporating the embeddings after the convolutional layer/block 3 mostly outperforms the operations on other layers/blocks. This can be caused by more discrimination power obtained by applying the pre-trained FluSense models.

From Table 6, we further validate the generalisation ability of the proposed CovNet with the DiCOVA Track-1 and ComParE CCS datasets. By competing with the official baselines, even simple CNNs can also achieve better performance with the proposed transfer learning methods. Therefore, the considered CovNet appears robust and can provide useful knowledge when detecting COVID-19 from crowd-sourced cough recordings. However, the performance improvement over the ComParE CCS baseline by incorporating the embeddings method is not obvious, which might be caused by the inherent data difference between the FluSense and COUGHVID datasets and the ComParE CCS dataset. Moreover, the CovNet works very well on the DiCOVA track-1 dataset, especially the incorporating embeddings. Perhaps, the embeddings from the pre-trained COUGHVID models carry more beneficial knowledge compared with parameters of convolutional layers on the DiCOVA dataset.

The main purpose of this study is to introduce and prove the usefulness of the transfer learning framework CovNet, instead of competing with the state-of-the-art performance on the DiCOVA Track-1 dataset (54–56) and ComParE CCS dataset (19). The constructed four CNN models are so simple that each of them only contains three convolutional layers/blocks; we do not apply any data augmentation techniques and the only input to the networks are the original log Mel spectrograms.

## 5. Conclusions and Future Work

In this study, we proposed a transfer learning framework, CovNet, containing transferring parameters and incorporating embeddings. Transferring parameters indicate fine-tuning the models by initialising and freezing some parameters with the pre-trained model; incorporating embeddings describe concatenating or adding the embeddings generated by a pre-trained model with the embeddings produced by the current model.

The effectiveness and generalisation ability of the proposed transfer learning framework was demonstrated when developing simple CNNs for COVID-19 detection from crowd-sourced cough sounds. In the future, one should consider deeper neural networks to further improve performance through transfer learning. Moreover, other knowledge transfer architectures, such as multi-task learning (57) and domain adaption (58) can be explored.

## Data Availability Statement

The original contributions presented in the study are included in the article/supplementary material, further inquiries can be directed to the corresponding author/s.

## Author Contributions

YC contributed to the study design, experimenting, manuscript drafting, and editing. XJ contributed to the experimenting and manuscript editing. ZR contributed to the study design, manuscript drafting, and editing. BS supervised the whole process, from study design, overall implementation, to manuscript drafting, and editing. All authors approved the submitted version.

## Funding

This study was partially supported by the Horizon H2020 Marie Skłodowska-Curie Actions Initial Training Network European Training Network (MSCA-ITN-ETN) project under grant agreement No. 766287 (TAPAS), the DFG's Reinhart Koselleck project No. 442218748 (AUDI0NOMOUS), and the Federal Ministry of Education and Research (BMBF), Germany under the project LeibnizKILabor (Grant No. 01DD20003).

## Conflict of Interest

The authors declare that the research was conducted in the absence of any commercial or financial relationships that could be construed as a potential conflict of interest.

## Publisher's Note

All claims expressed in this article are solely those of the authors and do not necessarily represent those of their affiliated organizations, or those of the publisher, the editors and the reviewers. Any product that may be evaluated in this article, or claim that may be made by its manufacturer, is not guaranteed or endorsed by the publisher.

## Footnotes

^1^https://www.who.int/emergencies/diseases/novel-coronavirus-2019/technical-guidance/naming-the-coronavirus-disease-(covid-2019)-and-the-virus-that-causes-it; retrieved 10 August 2021.

^2^https://covid19.who.int/; retrieved 10 August 2021.

^3^ https://zenodo.org/record/4498364#.YRKa3IgzbD4

^4^ https://github.com/ychang74/CovNet

^5^https://COUGHVID.epfl.ch/; retrieved 09 July 2021.

^6^https://www.covid-19-sounds.org/; retrieved 15 July 2021
